# Mosquito Feeding Assays to Determine the Infectiousness of Naturally Infected *Plasmodium falciparum* Gametocyte Carriers

**DOI:** 10.1371/journal.pone.0042821

**Published:** 2012-08-22

**Authors:** Teun Bousema, Rhoel R. Dinglasan, Isabelle Morlais, Louis C. Gouagna, Travis van Warmerdam, Parfait H. Awono-Ambene, Sarah Bonnet, Mouctar Diallo, Mamadou Coulibaly, Timoléon Tchuinkam, Bert Mulder, Geoff Targett, Chris Drakeley, Colin Sutherland, Vincent Robert, Ogobara Doumbo, Yeya Touré, Patricia M. Graves, Will Roeffen, Robert Sauerwein, Ashley Birkett, Emily Locke, Merribeth Morin, Yimin Wu, Thomas S. Churcher

**Affiliations:** 1 Department of Immunity and Infection, London School of Hygiene & Tropical Medicine, London, United Kingdom; 2 Department of Medical Microbiology, Radboud University Nijmegen Medical Centre, Nijmegen, The Netherlands; 3 W. Harry Feinstone Department of Molecular Microbiology and Immunology, Johns Hopkins Bloomberg School of Public Health and Malaria Research Institute, Baltimore, Maryland, United States of America; 4 Laboratoire de Recherche sur le Paludisme, Organisation de Coordination pour la lutte contre les Endémies en Afrique Centrale (OCEAC), Institut de Recherche pour le Développement, Yaoundé, Cameroun; 5 Maladies Infectieuses et Vecteurs : Écologie, Génétique, Évolution et Contrôle (MIVEGEC), Institut de Recherche pour le Développement, Montpellier, France; 6 Centre de Recherche et de Veille sur les Maladies Emergentes dans l'Océan Indien, La Réunion, France; 7 USC INRA Bartonella-tiques, Agence National de Sécurité Sanitaire, Maisons Alfort, France; 8 Malaria Research and Training Center, University of Sciences, Techniques and Technology, Bamako, Mali; 9 Malaria Research Unit of the Laboratory of Applied Biology and Ecology, University of Dschang, Dschang, Cameroon; 10 Department of Medical Microbiology, Microbiology Laboratory Twente, Enschede, The Netherlands; 11 School of Public Health-Tropical Medicine and Rehabilitation Sciences, James Cook University, Cairns, Queensland, Australia; 12 PATH Malaria Vaccine Initiative, Washington, D.C., United States of America; 13 Laboratory of Malaria Immunology and Vaccinology, National Institute of Allergy and Infectious Diseases, Rockville, Maryland, United States of America; 14 Infectious Disease Epidemiology-MRC Centre for Outbreak Analysis and Modelling, Imperial College London, London, United Kingdom; Tulane University, United States of America

## Abstract

**Introduction:**

In the era of malaria elimination and eradication, drug-based and vaccine-based approaches to reduce malaria transmission are receiving greater attention. Such interventions require assays that reliably measure the transmission of *Plasmodium* from humans to *Anopheles* mosquitoes.

**Methods:**

We compared two commonly used mosquito feeding assay procedures: direct skin feeding assays and membrane feeding assays. Three conditions under which membrane feeding assays are performed were examined: assays with i) whole blood, ii) blood pellets resuspended with autologous plasma of the gametocyte carrier, and iii) blood pellets resuspended with heterologous control serum.

**Results:**

930 transmission experiments from Cameroon, The Gambia, Mali and Senegal were included in the analyses. Direct skin feeding assays resulted in higher mosquito infection rates compared to membrane feeding assays (odds ratio 2.39, 95% confidence interval 1.94–2.95) with evident heterogeneity between studies. Mosquito infection rates in membrane feeding assays and direct skin feeding assays were strongly correlated (p<0.0001). Replacing the plasma of the gametocyte donor with malaria naïve control serum resulted in higher mosquito infection rates compared to own plasma (OR 1.92, 95% CI 1.68–2.19) while the infectiousness of gametocytes may be reduced during the replacement procedure (OR 0.60, 95% CI 0.52–0.70).

**Conclusions:**

Despite a higher efficiency of direct skin feeding assays, membrane feeding assays appear suitable tools to compare the infectiousness between individuals and to evaluate transmission-reducing interventions. Several aspects of membrane feeding procedures currently lack standardization; this variability makes comparisons between laboratories challenging and should be addressed to facilitate future testing of transmission-reducing interventions.

## Introduction

The transmission of malaria parasites begins with the presence of mature, sexual stage gametocytes in the peripheral blood of an infected individual. Once ingested by a blood feeding female *Anopheles* mosquito, male and female gametocytes become activated gametes that fuse to form zygotes. The zygotes develop into motile ookinetes which penetrate the mosquito midgut epithelium to form oocysts just beneath the basal lamina. The oocyst enlarges and matures over time and ruptures, after approximately 11–16 days for *P. falciparum* [1], to release sporozoites that migrate to the mosquito salivary glands. Once the sporozoites have invaded the salivary glands, the mosquito is infectious to humans. Parasite development in the mosquito can be negatively affected at each of these steps. As a consequence, not all gametocytes that are ingested by mosquitoes result in sporozoites in the salivary glands, and the transmission of malaria embodies more than just the epidemiology of gametocytes.

Our current understanding of the factors influencing transmission is far from complete. This may partially explain why the association between gametocyte density and mosquito infection rates is often described as tenuous or even non-existent [2], [3]. The infectiousness of gametocytes is known to be influenced by a large number of factors including their density [3], [4], [5], [6], sex-ratio [7], [8], the presence of transmission modulating factors such as antimalarial drug levels [9], [10], [11] and human and mosquito immune factors [12], [13], [14], [15]. Other elements that may influence gametocyte infectiousness include their maturity [16], [17] as well as poorly understood intrinsic parasite factors [18], [19].

**Figure 1:**
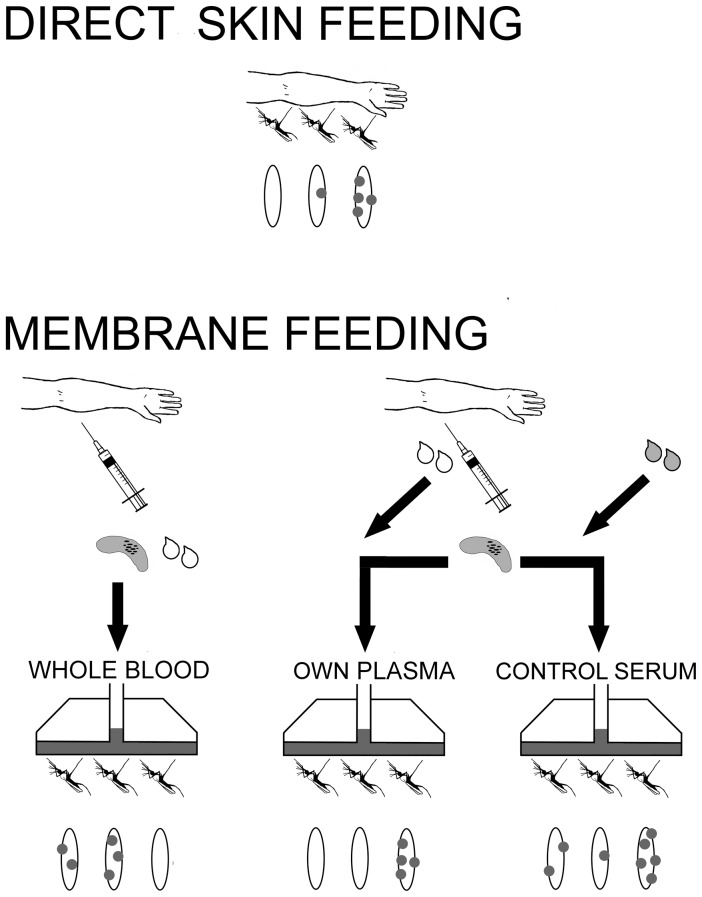
Mosquito feeding assays. In direct skin feeding assays, mosquitoes are placed directly on the skin of a parasitaemic host. In direct membrane feeding assays, a venous or finger prick blood sample from a naturally infected individual is offered to mosquitoes. Water-jacketed glass feeders are kept at approximately 37°C and mosquitoes feed through a membrane. In a whole blood assay, the blood sample is offered to mosquitoes directly. In a serum replacement experiment, the blood cells are offered to mosquitoes in a paired experiment in the presence of own plasma or of control serum.

The standard membrane feeding assay (SMFA) can be performed in the laboratory under controlled conditions to quantify the transmission-modulating effects of naturally acquired immune responses against cultured gametocytes [20], [21]. Studies that aim to quantify the human infectious reservoir or evaluate transmission reducing interventions such as gametocytocidal drugs or transmission-blocking vaccines require additional tools that can quantify the infectiousness of naturally infected gametocyte carriers to mosquitoes. Two mosquito feeding assays have been developed for this purpose. For direct skin feeding assays, mosquitoes are placed in direct contact with the skin of the gametocyte carrier and allowed to take a blood meal from skin microvasculature as they would naturally (Figure 1). For membrane feeding assays, a blood sample is offered to mosquitoes via an artificial feeder system. Membrane feeding assays can be conducted under three conditions: i) the blood meal can be added to the feeder immediately after sampling or after the blood pellet is separated from the plasma and resuspended in ii) autologous (own) plasma of the gametocyte carrier or iii) heterologous control serum [22] (Figure 1).

In this report, we compare mosquito infection rates in direct skin feeding and membrane feeding assays. In addition, we compare the different conditions used for membrane feeding assays, utilizing experiments conducted in five malaria endemic countries between 1996 and 2011. We use our findings, as well as communications with the researchers involved, to discuss the strengths and weaknesses of each assay and to identify shortcomings and areas for improvement in the current mosquito feeding procedures.

## Methods

We identified studies from the published literature that allowed a comparison between skin feeding and membrane feeding [23], [24], [25], [26], [27] and/or between different membrane feeding conditions [12], [28], [29], [30], [31], [32], [33]. The primary objective of many of these studies was to assess the human infectious reservoir for malaria [24], [26], [30], [33] or to study transmission-reducing immune responses [12], [28], [29], [30], [31], [32]; only three aimed to compare feeding assays or conditions directly [23], [25], [27]. Authors were contacted and asked to share the raw data underlying the published manuscripts. This resulted in seven datasets from Cameroon, The Gambia, Mali and Senegal. A dataset from Papua New Guinea [26] was shared by the authors but was unavailable for analysis due to technical problems with data retrieval from outmoded storage media. These data could therefore not be used in the statistical analysis but discussions with authors were included in our review of methodology. We also asked that all of the authors share other published or unpublished datasets that could facilitate our analysis and provide detailed information on the methodology that was used. Our request yielded additional unpublished data to augment datasets from Cameroon [23] and Mali [25], [34] and a previously unpublished dataset from Cameroon, using protocols described elsewhere [31], [35]. Data from a recent unpublished study from Cameroon was shared by the authors (Morlais, unpublished observations). This gave a total of ten studies with information on the methodology of feeding assays, of which nine provided raw data for statistical analysis. The individual studies received ethical clearance from the National Ethics Committee and Ministry of Public Health in Cameroon; the Institutional Ethical Committee of the National School of Medicine and Pharmacy in Mali; the Institute of Medical Research Institutional Review Board and the Papua New Guinea Medical Research Advisory Committee in Papua New Guinea; the review board of the Ministry of Health in Senegal; studies in the Gambia received ethical clearance from the joint Medical Research Council/Gambia Government Joint Ethical Committee and from the London School of Hygiene & Tropical Medicine ethics committee. Written informed consent was obtained from participants and/or their parent(s) or guardian(s).

### Statistical analysis

Statistical analyses were done in Stata software (version 10; Statacorp) and in R version 2.10.0. The percentage of mosquitoes that became infected was compared using a Bland Altman plot [36] where the difference in the proportion of infected mosquitoes between two feeding conditions (e.g. for one individual experiment the proportion of infected mosquitoes in skin feeding – the proportion of infected mosquitoes in whole blood membrane feeding) was plotted against the mean proportion of infected mosquitoes (e.g. for one individual experiment the total number of infected mosquitoes in skin feeding and whole blood membrane feeding combined/total number of examined mosquitoes in skin feeding and whole blood membrane feeding combined). The Wilcoxon signed-rank test for matched pairs was used for pairwise comparisons of the different assays using blood from the same donor. Because the number of mosquitoes dissected varied between experiments, 95% confidence intervals were estimated using bootstrapping methodology to illustrate the uncertainty generated by the different sampling efforts. A random-effects meta-analysis [37] was used, which generated odds ratios (OR) for the matched pairs, allowing for a comparison of the overall effectiveness (i.e. the proportion of infected mosquitoes) of the different assays or conditions. This analysis was restricted to experiments that were considered successful, defined as an experiment with different feeding conditions for which at least one mosquito was infected in one (but not necessarily all) feeding conditions. In order to allow all feeding conditions of successful experiments to contribute to the overall OR estimates, assay conditions which failed to infect any mosquitoes were given a value of half the detection threshold of infected mosquitoes (0.5 divided by the number of mosquitoes dissected) and included in the analysis. This approach is in line with common meta-analysis procedures [37]. Experiments where no mosquitoes were infected in any of the conditions were considered experiment failures and excluded from this analysis. The difference in the number of successful experiments and infected mosquitoes in feeds with or without detectable gametocytes in the sample was determined by chi-square test. The correlation between gametocyte density as continuous variable and mosquito infection rates was determined by Spearman correlation.

## Results

### Study characteristics and procedures

An overview of each of the study populations is given in Table 1. Participants for membrane feeding assays were recruited during community surveys, at health facilities among attendees or in randomized clinical trials. Most studies had a minimum age of recruitment, typically ≥2 or ≥3 years with the exception of the study Cameroon-3 and experiments from The Gambia that also enrolled younger children. For direct skin feeding assays, a minimum age of 4–5 years was imposed in three studies [23], [25], [27]. This limit was set based on a consensus among scientists and local health authorities to minimise discomfort in younger individuals. Individuals were recruited based on microscopically detectable gametocytes at the time of membrane feeding; gametocytes were detected by screening 100–200 microscopic fields or by examining microscopic fields until 300–3,000 leukocytes were seen (Table 1). One study also purposefully included individuals without microscopically detectable gametocytes to ‘define the threshold of infectiousness’ [23]; 52.2% (12/23) of these were microscopically free of both gametocytes and asexual parasites. Several others used an initial slide per participant to select only those who were gametocytemic for the experiment. Some of these selected individuals were gametocyte negative on a second slide that was prepared at the time of feeding, illustrating that gametocytes frequently circulate at densities around the threshold for microscopic detection [22] and vary from day to day.

**Table 1:** Overview study populations.

Country	Year	Population	Age range or minimum age (years)	Gametocyte screening	Gametocyte prevalence in selected individuals (n/N)	Median gametocyte density (IQR)
Cameroon-1 [30]	1993	Clinic attendees	4–63	1000 WBC	97.6 (121/124)	156 (72–400)
Cameroon-2 [23]	1996–7	Survey participants	≥5	3000 WBC	82.6 (38/46)	23 (6–70)
Cameroon-3 [29]	1996	Clinic attendees	1–63	1000 WBC	98.2 (55/56)	296 (88–536)
Cameroon-4 [33]	1996–8	Survey participants	≥2	3000 WBC	95.8 (69/72)	36 (12–128)
Cameroon-5 (Gouagna *et al.*, unpublished)	1998	Survey participants & clinic attendees	≥2	500 WBC	100 (22/22)	275 (78–456)
Cameroon-6 (Morlais *et al.*, unpublished)	2011	Survey participants	5–11	1000 WBC	100 (25/25)	24 (21–36)
Mali [25], [34]	1996–8	Survey participants	4–18	300 WBC	97.1 (68/70)	100 (50–225)
Papua New Guinea [26]	1983–1985	Survey participants & clinic attendees	0.5–≥20	100 fields	NA	NA
Senegal [27]	1998	Survey participants	6–57	200 fields	100 (60/60)	269 (91–562)
The Gambia [17], [28], [38], [39]	1998–2000	Confirmed malaria cases	0.5–17	100 fields	85.1 (292/343)	56 (15–280)

Year = year of data collection; Survey participants = participants of cross-sectional surveys, largely asymptomatic population, were screened for gametocytes; clinic attendees = individuals with suspected malaria were screened for gametocytes; n = number of gametocyte positive individuals; N = number of people screened for gametocytes among the study population; gametocyte density = median density/µL for gametocyte positive individuals only; IQR = interquartile range (25^th^ and 75^th^ percentile), WBC = white blood cells.

All studies used 3–5 day old adult female *Anopheles gambiae s.s.* or *Anopheles arabiensis* mosquitoes. In studies in The Gambia and Mali the next generation progeny of wild-caught gravid female mosquitoes were used; studies in Senegal collected mosquitoes at larval stages from breeding sites; studies in Cameroon used locally founded and reared laboratory colony mosquitoes that were adapted to feeding on a membrane feeder (Table 2).

**Table 2:** Overview mosquito feeding procedures.

Country	Timing of membrane feeding	Mosquito species	Mosquito origin	Membrane	Mercuro-chrome	Mosquitoes dissected per experiment, median (IQR)				Number of feeds (infected)			
						skin	whole	own	control	skin	whole	own	control
Cameroon-1 [30]	Enrolment	*An gambiae s.s. M form*	Colony	Parafilm®	2%	ND	30(28–30)	30(23–30)	29(21–30)	ND	140 (75)	110 (47)	106 (61)
Cameroon-2 [23]	Enrolment	*An gambiae s.s. M form*	Colony	Parafilm®	3%	27(18–30)	29(17–30)	ND	ND	47 (27)	34 (24)	ND	ND
Cameroon-3 [29]	Enrolment	*An gambiae s.s. M form*	Colony	Parafilm®	2%	ND	30(29–30)	29(26–30)	29(24–30)	ND	58 (50)	58 (39)	58 (57)
Cameroon-4 [33]	Enrolment	*An gambiae s.s. M form*	Colony	Parafilm®	2%	30(27–30)	30(26–30)	29(24–30)	29(23–30)	55 (55)	39 (39)	30 (30)	31 (31)
Cameroon-5 (Gouagna *et al.*, unpublished)	Enrolment	*An gambiae s.s., M form*	Colony	Parafilm®	2%	21(15–25)	20(20–25)	ND	ND	24 (17)	22 (13)	ND	ND
Cameroon-6 (Morlais *et al.*, unpublished)	Enrolment	*An gambiae s.s. M form*	Colony	Parafilm®	0.4%	ND	57(43–70)	54(43–60)	56(47–64)	ND	25 (22)	24 (22)	25 (25)
Mali [25], [34]	Enrolment	*An gambiae s.l.*	F1 progeny	Parafilm®	0.5%	78(51–92)	75(58–116)	ND	ND	70 (59)	70 (54)	ND	ND
Papua New Guinea [26]	Enrolment	*An farauti*	Colony	Baudruche®	0.2%	NA	NA	ND	ND	NA	NA	ND	ND
Senegal [27]	Enrolment	*An. arabiensis*	Larval collections	Baudruche®	1%	14(10–24)	6(3–10)	ND	ND	60 (25)	56 (17)	ND	ND
The Gambia [17], [28], [38], [39]	After treatment	*An. gambiae s.l.*	F1 progeny	Parafilm®	0% or 2%	ND	ND	18(13–23)	19(13–23)	ND	ND	348 (95)	366 (129)

IQR = interquartile range (25^th^ and 75^th^ percentile). Skin = direct skin feeding; whole = membrane feeding with whole blood sample; own = membrane feeding with serum replacement, red blood cells are re-suspended in the donor's own (autologous) plasma; control = membrane feeding with serum replacement, red blood cells are re-suspended in malaria-naïve control serum; infected = at least one infected mosquito; NA = not available; ND = not done.

### Procedures in direct skin feeding assays

Direct skin feeding assays were performed in Cameroon, Mali and Senegal. Boxes or paper cups with a total of 60–70 mosquitoes were applied to the inner thigh (Cameroon-2 & 4, [23]) or calves (Senegal, [27], Mali, [25]), and were allowed to feed for 10–15 minutes [23], [27]. All studies provided anti-histamine cream to the volunteer after the experiment to reduce inflammation caused by mosquito bites. Unfed mosquitoes were discarded and only fully engorged mosquitoes were transferred to rearing containers and maintained in the insectary at temperatures and relative humidity in the range of 25–28°C and 70–90%, respectively. On day 7–8 after feeding, surviving mosquitoes were dissected in 0.5–3% mercurochrome in phosphate buffered saline (PBS) or distilled water (Table 2).

### Procedures in membrane feeding assays

In membrane feeding assays, venous blood was taken in citrate–phosphate dextrose (The Gambia, Mali) or heparin (Cameroon, Senegal) using syringes that were pre-warmed in 37°C incubators. In the study from Papua New Guinea, 200 µL finger prick blood samples were drawn into heparinised containers [26]. Whole blood membrane feeding experiments were conducted with finger prick and venous blood samples; serum replacement experiments were only performed with venous blood samples. For whole blood membrane feeding experiments, blood samples were offered to mosquitoes immediately without sample modulation (WHOLE BLOOD). For serum-replacement experiments in The Gambia, the samples were centrifuged for 5 minutes, and the plasma was removed. After being washed with pre-warmed medium (Roswell Park Memorial Institute, RPMI) [29], the red blood cell pellet was split into two aliquots of 300–500 µL each. These were resuspended to a packed cell volume of 33% in either the original plasma (OWN PLASMA) or in pooled AB serum from European donors with no history of malaria exposure (CONTROL SERUM). In studies from Cameroon, the blood sample was centrifuged for 3–5 minutes after which the original plasma was removed. No washing procedure took place; equal volumes of the red blood pellet were resuspended in either the original autologous plasma (OWN PLASMA) or in pooled malaria naïve AB serum (CONTROL SERUM). In order to keep all materials at approximately 37°C during the experiment, temperature-controlled centrifuges were used in some experiments [17], [28], [38], [39] while others placed a centrifuge inside a heated incubator or cabinet [23], [29], [31]. In all studies, starved mosquitoes were allowed to feed for 15–30 min via an artificial membrane (Parafilm or Baudruche) attached to a water-jacketed glass feeder to maintain the temperature at approximately at 37°C. After feeding, unfed mosquitoes were removed. Blood-fed mosquitoes were kept at a temperature range of 26 to 28°C with permanent access to a 10% sucrose solution without further blood meals. Mosquito midguts were dissected 7–8 days later in PBS (The Gambia) or 0.2% (Papua New Guinea) 0.4% (Cameroon), 0.5% (Mali), 1% (Senegal) or 2% (Cameroon, The Gambia) mercurochrome in PBS or distilled water. The number of oocysts on the mosquito midgut was recorded.

### Outcomes of mosquito feeding assays

We analysed a total of 930 experiments from nine studies wherein mosquitoes were dissected on day 7–8 post-blood feeding. The proportion of successful experiments, defined as at least one mosquito becoming infected after feeding in any of the experimental conditions, was 62.0% (577/930) but varied widely between studies: 44.8% (165/368) in The Gambia, 48.9% (44/90) in Cameroon-2, 51.7% (31/60) in Senegal, 56.0% (79/141) in Cameroon-1, 85.7% (60/70) in Mali, 87.5% (21/24) in Cameroon-5, 100% (58/58) in Cameroon-3, 100% (94/94) in Cameroon-4 and 100% (25/25) in Cameroon-6.

### Comparison of infection outcomes between skin feeding and whole blood membrane feeding

**Figure 2:**
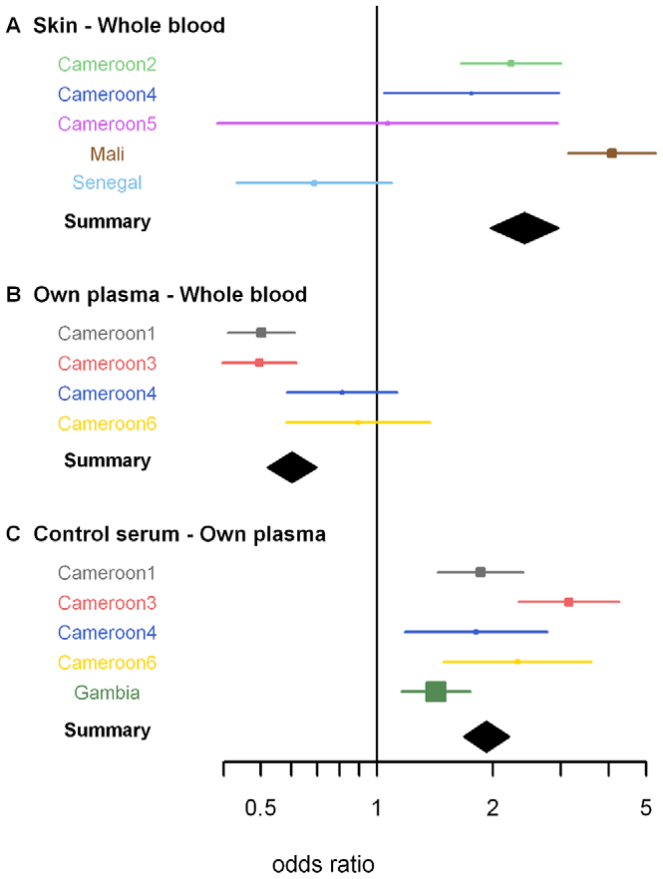
A forest plot showing the overall result of each study together with the overall estimates of the meta-analysis. All data were combined to generate the summary odds ratio and 95% confidence interval estimates denoted by the black diamond. Odds ratios are calculated with the latter feeding condition as reference; e.g. for Figure 2A the odds ratio for mosquito infection in skin feeding experiments is calculated with whole blood membrane feeding as reference. Points are weighted according to the number of paired experiments in the study (indicated by the size of the data point).

**Figure 3:**
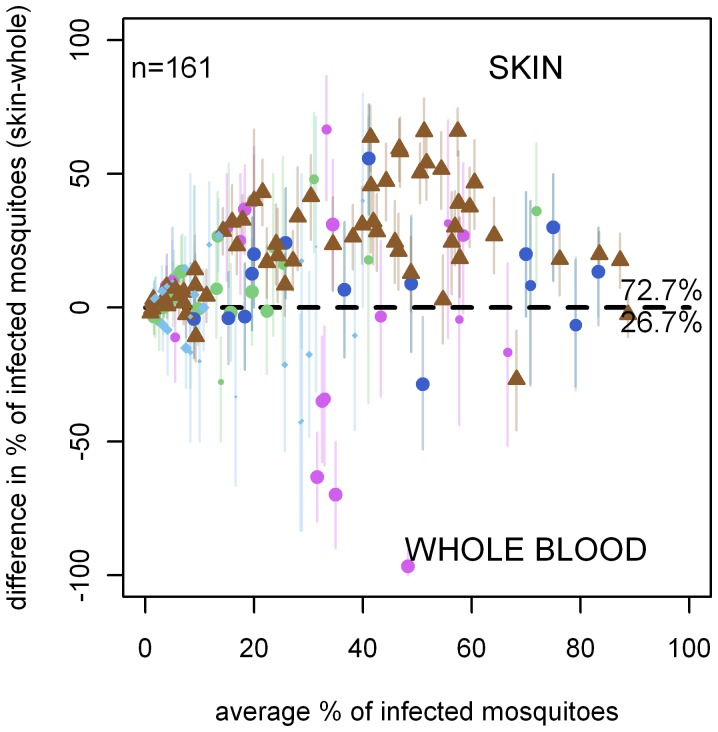
Skin feeding versus membrane feeding. Results are from 161 paired experiments plotted in a Bland-Altman plot. Points above the line had higher infectivity in the skin feeding assay than the whole blood SMFA. Point colour denotes the study using the same key as in Figure 2. The shape of the point denotes the country the experiment was carried out in, be it Cameroon (circle), Mali (triangle) or Senegal (diamond). The size of the points indicates the relative average number of mosquitoes dissected in the experiment, with points>50 mosquitoes dissected having the same size). In 117 experiments (72.7%), the proportion of infected mosquitoes was higher in skin feeding assays compared to the paired whole blood membrane feeding assay; in 43 experiments (26.7%) the opposite was observed.

**Figure 4:**
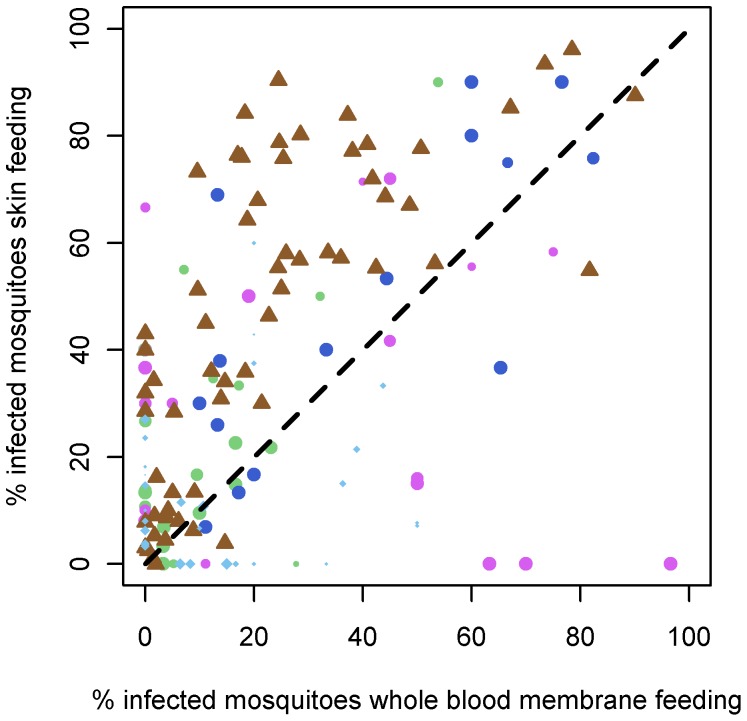
The association between mosquito infection rates in skin feeding experiments and whole blood membrane feeding assays. The proportion of infected mosquitoes in whole blood membrane feeding assays (X-axis) is strongly associated with the proportion of infected mosquitoes in skin feeding assays (Spearman's rho 0.36, p<0.0001). Point size, shape and colour are as described in Figure 3. The shape of the point denotes the country the experiment was carried out in, be it Cameroon (circle), Mali (triangle) or Senegal (diamond).

The dataset contained 241 paired direct skin feeding and whole blood membrane feeding experiments, 66.8% (161/241) of which resulted in at least one infected mosquito. When all experiments were considered together, the odds ratio for mosquito infection in direct skin feeding compared to whole blood membrane feeding was 2.39 (95% CI 1.94–2.95) (Figure 2A). The outcomes of individual paired experiments are given in Figure 3. We observed that a pairwise comparison of feeding conditions indicated a higher proportion of infected mosquitoes in direct skin feeding compared to membrane feeding assays (p<0.0001). If the studies were examined separately, there was a significantly higher proportion of infected mosquitoes by skin feeding in Cameroon-2 (p = 0.0001), Cameroon-4 (p = 0.004) and Mali (p<0.0001) but not in Cameroon-5 (p = 0.88) or Senegal (p = 0.35). Despite the higher infection rates in skin feeding experiments, there was a strong positive association between the infection rates by membrane feeding and by skin feeding in successful experiments (Spearman rho = 0.33, p<0.0001; Figure 4). This was also true when studies were considered separately (Cameroon-2, p<0.001; Cameroon-4, p = 0.004; Mali, p<0.0001) but not for Cameroon-5 (p = 0.55) and Senegal (p = 0.76).

### Comparison of infection outcomes between serum replacement and whole blood membrane feeding assays

**Figure 5:**
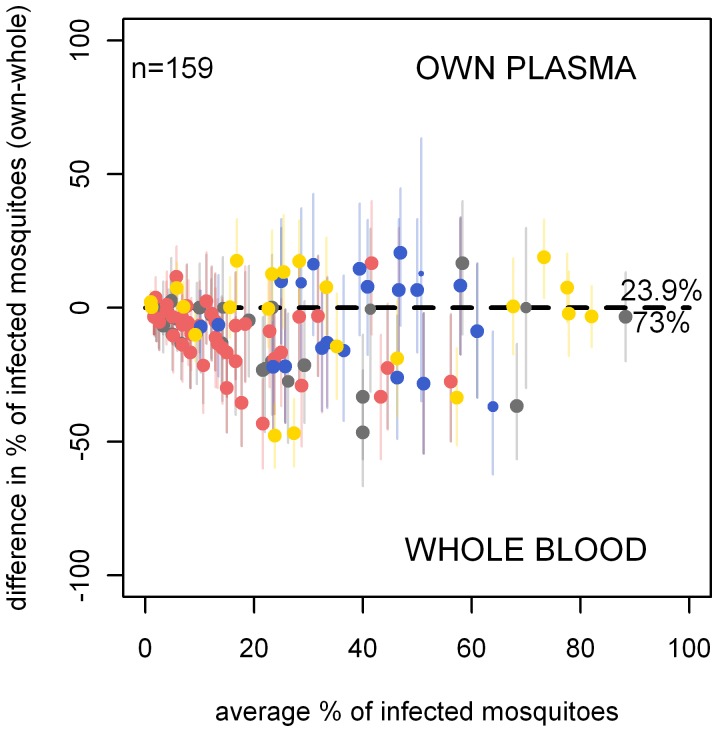
Membrane feeding with whole blood and with own plasma. The results are from 159 paired experiments. In 116 experiments (73.0%), the proportion of infected mosquitoes was higher in experiments with whole blood samples (i.e., without sample modulation) compared to serum replacement experiments where the blood pellet went through additional manipulation before being re-suspended with the gametocyte donor's own plasma. In 38 experiments (23.9%) the opposite was observed. Point size, shape and colour are as described in Figure 3. All experiments were performed in Cameroon.

There were 212 membrane feeding experiments where one blood sample was split and one aliquot was used in serum replacement experiments while the other was offered to mosquitoes directly. These experiments, of which 63.7% (161/212) resulted in at least one infected mosquito, allow a comparison of two membrane feeding conditions that only differ in sample handling procedures. In whole blood experiments, red blood cells and plasma are offered to mosquitoes immediately; in serum replacement experiments with own plasma the exact same material is offered to mosquitoes after centrifugation. When all of these experiments were considered together, the odds ratio for mosquito infection in membrane feeding with own plasma compared to whole blood membrane feeding was 0.60 (95% CI 0.52–0.70)(Figure 2B). The outcomes of individual paired experiments are given in Figure 5. A pairwise comparison of feeding conditions indicated a lower proportion of infected mosquitoes in experiments where red blood cells were resuspended with own plasma after centrifugation compared to experiments where the whole blood sample was offered to mosquitoes immediately (p<0.0001). When studies were considered separately, this lower infection rate in serum replacement experiments compared to whole blood membrane feedings was apparent in Cameroon-1 (p<0.001) and Cameroon-2 (p<0.001), studies conducted in 1996–1997. There was no statistically significant difference in the proportion of infected mosquitoes between the two assay conditions in Cameroon-4 (1996–1998; p = 0.31) and Cameroon-6 (2011; p = 0.85). The documented methodology of all these studies was identical, although unrecorded differences in sample handling procedures or equipment cannot be ruled out.

### Comparison of infection outcomes in serum replacement experiments

**Figure 6:**
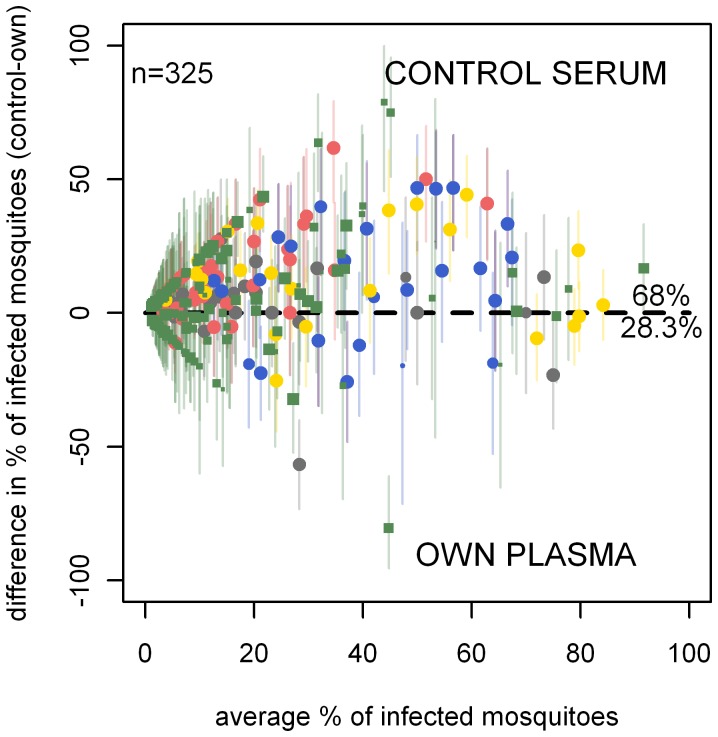
Membrane feeding with and without serum replacement. The results are from 325 paired experiments. In 221 experiments (68.0%), the proportion of infected mosquitoes was higher in experiments with malaria naïve control serum compared to the gametocyte donor's own plasma; in 92 experiments (28.3%) the opposite was observed. The shape of the point denotes the country the experiment was carried out in, be it Cameroon (circle) or The Gambia (square). Point size, shape and colour are as described in Figure 3.

A total of 557 membrane feeding assays with serum replacement (own plasma versus control serum) were done, 58.7% (327/557) of which resulted in at least one infected mosquito. When all these experiments were considered together, the odds ratio for mosquito infection in membrane feeding with control serum compared to own plasma was 1.92 (95% CI 1.68–2.19)(Figure 2C). The outcomes of individual paired experiments are given in Figure 6. Pairwise comparison of feeding conditions indicated a higher proportion of infected mosquitoes in experiments after serum replacement compared to own autologous plasma (p<0.0001). If studies were considered separately, a higher proportion of infected mosquitoes after serum replacement was evident for all studies: Cameroon-1 (p<0.0001), Cameroon-3 (p<0.0001), Cameroon-4 (p = 0.003), Cameroon-5 (p = 0.003)), Cameroon 6 (p = 0.002) and The Gambia (p = 0.002).

### The association between gametocyte density and mosquito infection rates

**Figure 7:**
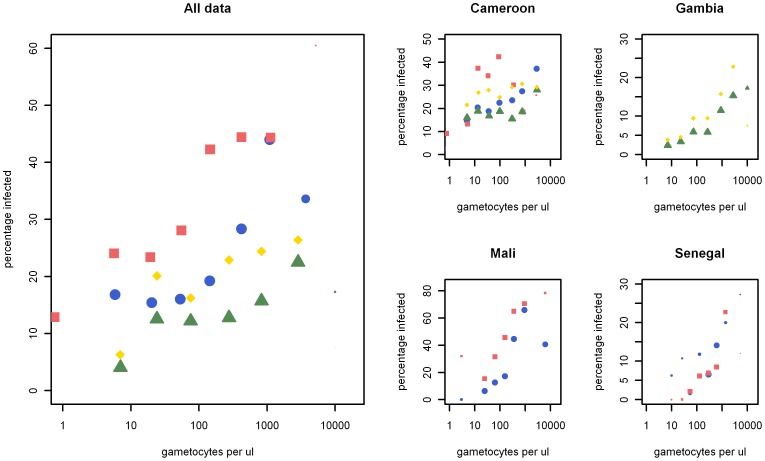
The association between gametocyte density and mosquito infection rates for different feeding conditions. Plotted is the association between gametocyte density and mosquito infection rates (oocyst prevalence) for skin feeding assays (red squares), whole blood membrane feeding assays (blue circles) and serum replacement experiments with own plasma (green triangles) and control serum (yellow diamonds). Data were grouped according to gametocyte density estimates into 8 equally sized bins (on a log scale). Panel A shows all the data grouped together whilst B to E show the data grouped by country. The size of the data points is relative to the number of mosquitoes dissected in each bin.

The proportion of successful experiments (i.e. in which at least one mosquito was infected in any feeding condition) was 66.0% (510/773) for experiments where the donor had gametocytes detected by microscopy at the time of feeding compared to 27.6% (24/87) in experiments where the slide taken on the day of feeding was gametocyte negative by microscopy (Table 3, p<0.0001). The proportion of infected mosquitoes was 20.9% (9,952/47,590) for microscopically confirmed gametocyte carriers compared to 4.9% (184/3,731) for individuals without gametocytes detected by microscopy (p<0.0001). There was a positive association between microscopically determined gametocyte density and the proportion of infected mosquitoes in skin feeding assays (p = 0.0001), whole blood membrane feeding assays (p<0.0001) and membrane feeding assays with own plasma (p<0.0001) or control serum (p<0.0001) (Figure 7).

**Table 3:** The proportion of successful experiments and the proportion of infected mosquitoes in relation to gametocyte density in the feed sample.

Gametocyte density per µL	Proportion successful experiments	Proportion of infected mosquitoes
0	27.6 (24/87)	4.9 (184/3,731)
1–25	57.9 (121/209)	14.0 (1,586/11,307)
26–50	70.2 (73/104)	14.2 (951/6,674)
51–100	59.3 (64/108)	20.6 (1,557/7,557)
101–250	63.6 (82/129)	25.4 (2,224/8,743)
251–500	75.3 (73/97)	27.3 (1,699/6215)
>500	77.0 (97/126)	27.3 (1,925/7,094)
Gametocytes at all densities	66.0 (510/773)	20.9 (9,952/47,590)

## Discussion

We described membrane feeding procedures used in five different countries and analyzed data from 930 mosquito feeding experiments on naturally infected individuals from West and Central Africa. Using this unique dataset, we present evidence that direct skin feeding experiments result in higher mosquito infection rates than membrane feeding experiments although the outcomes in both assays are strongly correlated. Furthermore, when the serum of malaria-infected individuals was replaced with malaria-naïve control serum prior to feeding, feedings result in higher mosquito infection rates. However, the sample processing involved in serum replacement experiments may result in a loss in parasite transmission efficiency.

### The efficiency of direct skin feeding and membrane feeding assays

When we analysed the dataset as a whole, we found clear indications that the proportion of infected mosquitoes is higher in direct skin feeding assays compared to whole blood membrane feeding. Looking at the datasets individually, three of five studies showed a significantly higher proportion of infected mosquitoes after direct skin feeding while two out of five individual studies did not. One of the studies that did not show a difference in mosquito infection rates between direct skin feeding and membrane feeding was a relatively small study from Cameroon (22 experiments), which may have provided too few data points to show significant difference. The other was a larger study from Senegal (141 experiments) where the number of examined mosquitoes per experiment was the smallest of all studies we considered. The median number of examined *An. arabiensis* mosquitoes in this study was 14 (Inter-quartile range (IQR) 10–24) in the direct skin feeding experiments and 6 (IQR 3–10) in the membrane feeding assays. By comparison, the median number of examined *An. gambiae* mosquitoes was 76 (IQR 51–116) for the study in Mali where the difference in mosquito infection rates between direct skin feeding and membrane feeding was evident. Considering the association between the proportion of examined mosquitoes and the proportion of successful experiments, it is possible that the inter-study variation in pairwise comparisons of direct skin feeding and membrane feeding is a consequence of differences in the number of examined mosquitoes and thereby the precision of studies.

The lower sensitivity of membrane feeding assays to detect infectious individuals may reflect technical shortcomings of this assay or differences in gametocyte concentrations between the blood meal sources. During the membrane feeding assay, the infectiousness of gametocytes may be affected such that gametocytes can become activated in the feeder prior to ingestion by mosquitoes [5]. This activation is at least partly mediated by a drop in temperature below 37°C. If activation occurs prior to engorgement by mosquitoes, gametocyte infectiousness drops dramatically, highlighting the necessity to keep blood samples at 37°C at all times from blood draw through to the end of feeding. This also argues against the use of finger prick blood samples for membrane feeding since the sampling time, during which temperature is difficult to control, is likely to be longer for finger prick sampling than for venous sampling.

Alternatively, or in addition to a loss of gametocyte infectiousness due to gametocyte activation, a higher proportion of infected mosquitoes in direct skin feeding assays may reflect differences in gametocyte concentration or maturity in different parts of the blood vascular system. Gametocyte concentrations may be higher in the microvasculature of the skin compared to finger prick or venous blood samples [40], [41], possibly related to mechanical properties of *P. falciparum* gametocytes [42] or currently unknown gametocyte aggregation [43] or gametocyte-endothelial adhesion mechanisms [44]. The concentration of gametocytes in the microvasculature requires comparative analyses and confirmation using the currently available molecular gametocyte detection techniques [45] that preferably differentiate between different developmental stages of gametocytes [46].

### Strengths and weaknesses of membrane feeding assays

We found a significant correlation between the outcomes of direct skin feeding assays and whole blood membrane feeding assays [23], [25]. Although the predictive value of a single pairwise comparison might be relatively poor (Figure 4), pooling data from multiple feeding experiments gives good agreement between the two assays. As such, direct skin feeding experiments may give the most sensitive estimate of the human infectious reservoir while membrane feeding assays are suitable tools to compare the infectiousness between individuals and to evaluate transmission-reducing interventions. Membrane feeding assays have advantages over direct skin feeding assays that include a wider ethical and practical acceptability, notably in the youngest age groups. Membrane feeding also avoids the risk of infection of study participants with pathogens that might be present in anophelines [47], although this risk may be negligible in anophelines that were raised in colony or newly emerged from field-collected larvae. Membrane feeding assays also allow a direct quantification of the gametocyte concentration in the blood meal source (Table 4) and the assessment of transmission reducing activity of serum components. We examined 557 paired membrane feeding experiments from 5 studies where gametocytes were offered to mosquitoes together with plasma of the gametocyte donor and control serum. In all studies, we found convincing evidence that replacing the gametocyte donor's serum with malaria naïve control serum increases the proportion of infected mosquitoes [12]. In several of the original studies the transmission reducing effect of the gametocyte donor's serum was related to the presence of antibody responses to sexual stage malaria antigens Pfs230 and Pfs48/45 [28], [29], suggesting a role for naturally acquired transmission reducing immune responses. Antimalarial drug levels may also affect mosquito infection rates [9], [10], [11] although we consider it unlikely that residual drug levels completely explain the higher infection rates after serum replacement since recent drug treatment was unrelated to mosquito infection rates in several of the original studies [12], [27], [30] and the existence of naturally acquired transmission reducing immunity has been confirmed in SMFA experiments where cultured gametocytes were repeatedly offered to mosquitoes in the presence of endemic sera [21].

**Table 4:** Strengths and weaknesses of different assays.

	Direct skin feeding	Membrane feeding	
		Whole blood	Serum replacement
**Advantages**	1. closely resembles natural feeding	1. direct assessment of gametocyte concentration	1. all advantages listed under ‘whole blood’
	2. no delay between sampling and mosquito feeding	2. repeated assays with large mosquito numbers are possible	2. potential effects of drugs, and transmission blocking blood components can be reduced or eliminated
	3. no requirement for venipuncture of volunteers	3. larger volunteer age-range acceptable	
**Disadvantages**	1. no direct assessment of gametocyte concentration	1. venous blood samples may have different gametocyte concentrations from the natural biting site	1. venous blood samples may have different gametocyte concentrations from the natural biting site
	2. potential effects of drugs, and transmission blocking blood components for interfering with interpretation of results	2. potential effects of drugs, and transmission blocking blood components for interfering with interpretation of results	2. sample manipulation requirements extend time between venipuncture and feeding and risk early gametocyte activation if 37°C temperature is not maintained for the blood samples
	3. ethically and practically less acceptable and feasible in young children	3. handling time between venipuncture and feeding risks early gametocyte activation if 37°C temperature is not maintained for the blood samples	
	4. potential for volunteers to be exposed to mosquito pathogens		
	5. not acceptable for ethics committees in some endemic countries		

Our analysis also allowed us to examine whether the serum replacement procedure affects the infectiousness of gametocytes. This aspect of membrane feeding assays has never been studied in detail. The experimental design to determine transmission reducing immunity in the field would ideally allow serum replacement without affecting the infectiousness of gametocytes during the replacement procedure. We observed strong evidence for a lower proportion of infected mosquitoes in the serum replacement experiment compared to whole blood membrane feeding assays in two studies [23], [29], while this effect was not apparent in two other studies [31] (Morlais *et al.* unpublished). This contrast raises questions about differences in methodology between the different studies.

### Variation in efficiency and procedures of feeding experiments

The experiments that were analysed in this manuscript were performed over a 15-year period and involved different research groups. It is therefore expected that there is variation in methodology. Standardization of procedures would be desirable going forward in order to prepare mosquito feeding experiments for clinical trials that, in the case of community trials for transmission blocking vaccines, are likely to involve multiple field sites and research centers [48]. Currently, the SMFA is the gold standard for the assessment of transmission reducing activities of sera in the laboratory. In the SMFA, unlike the mosquito feeding experiments described in this manuscript, gametocyte concentrations can be synchronized and standardized and quality control experiments can be used to minimize variability [20]. Mosquito feeding assays in the field, required to assess the human infectious reservoir and for the field evaluation of transmission reducing interventions under natural conditions, currently lack similar quality control mechanisms. This may explain the considerable variation in the proportion of successful feeds between studies, which ranged from 45–100% and highlights a gap between laboratory and field assays that needs to be bridged.

The membrane feeding experiments from the field that we considered for this report differed in their methodology. Distinct differences that could be gathered from the published reports include methods for gametocyte quantification, procedures related to serum replacement (e.g. centrifuge temperature, speed and duration of spinning, washing step, adjustment for hematocrit), species and source of mosquitoes, type of membrane, number of mosquitoes examined and the staining solution for oocyst detection. Differences between patient populations could also play a role: serum factors associated with clinical disease [49] and lower packed cell volume [50] may both reduce gametocyte infectiousness. A number of other potential differences are not typically recorded. Some of these differences were only mentioned during direct communication from researchers, and include time between venipuncture and membrane feeding, time that mosquitoes are allowed to feed on the membrane, and procedures to increase mosquito feeding rates (which include breathing close to the feeder or covering the feeder with materials with human odor, e.g. worn socks). Based on the complex nature of the feeding assays and the number of factors that could impact successful results, these latter differences may actually significantly impact results. In Table 5, we summarize our recommendations to maximize the success rates of mosquito feeding experiments and to answer some of the questions that are currently outstanding about the nature of malaria transmission. Some of these recommendations are based on the current analysis, others are based on unpublished consensus between the researchers who shared their data for the current report.

**Table 5:** Recommendations to maximize success rates and informativeness of mosquito feeding experiments.

1. Ensure procedures are in place to maintain samples at 37°C at all times from venipuncture to the end of the membrane feeding experiment, in order to prevent activation of gametocytes and reduction of infectivity.
2. Use commercially available membranes that are more amenable to standardization than animal skin.
3. Set an appropriate the time range that mosquitoes are allowed to feed on a blood meal. This is a purely ethical issue in direct skin feeding assays but may affect the validity of membrane feeding assays since the infectiousness of gametocytes may decline during membrane feeding experiments. A feeding time of 15 minutes is commonly used.
4. Keep mosquito number/cup size/age of mosquitoes and also mosquito husbandry in feeding experiments as constant as possible.
5. Remove unfed mosquitoes after the feeding experiment (rather than selecting fed mosquitoes).
6. Estimate the assay variability by repeating the same experiment multiple times and incorporate this information in power calculations and statistical analysis.
7. Track the number of dead mosquitoes prior to examination to determine differences in mosquito survival between cages/cups or between gametocyte donors.
8. Maximize the number of mosquitoes that can be used in experiments to ensure enough are dissected for achieving sufficient statistical power to address research questions.

### Gaps in our current understanding

In light of the variability in results identified in our analysis, there are some outstanding questions that need to be answered to gain a better understanding of the mosquito feeding assays (Table 6). On the parasite side, implementation of a reliable detection method for mature gametocytes is essential to further advance the field of malaria transmission research. Tools to differentiate between male and female gametocytes and between different developmental stages would be highly valuable. Availability of such tools could facilitate further study of the reasons for the apparent difference in mosquito infection rates between direct skin feeding and membrane feeding. Inconsistent mosquito infection rates could be addressed by better understanding the fitness and vector competence of the *Anopheles* mosquitoes used in the experiments. Mosquito husbandry practices may have an impact on the health and physiology of the vector [51], [52], [53]. Despite the growing body of evidence regarding mosquito innate immunity and the influence of the midgut microbiome on parasite development in the mosquito, more work is needed to better understand the importance of controlling the mosquito variable.

**Table 6:** Outstanding questions.

**Gametocyte**	Are there differences in gametocyte concentration or infectiousness between different blood compartments (e.g. microvasculature versus vein)?
	The following tools are desirable to answer these questions:
	1. reliable gametocyte quantification in the mosquito blood meal
	2. markers of gametocyte infectiousness
	3. sensitive tools to determine gametocyte sex-ratios
**Mosquito vector**	Does the variable permissibility of mosquito vectors for infection affect the outcomes of mosquito feeding assays?
	How do mosquito fitness factors influence the outcomes of mosquito feeding assays?
	Can gametocyte density influence mosquito survival rates?
**Feeding assay**	Can quality control measures be included in feeding assays conducted under field conditions?
	Can the serum replacement procedure be optimised to minimize/prevent a loss in gametocyte infectiousness?
	The following tools are desirable for this purpose:
	1. a standardized protocol across field sites
	2. a positive control for mosquito feeding experiments in the field
	3. a high throughput system to sensitively detect oocysts in individual or pooled mosquitoes

The source of mosquitoes may also require further study. When using mosquito colonies, mosquitoes can be reared in a controlled manner to improve the robustness and reproducibility of assays and select for aggressive blood feeding through a membrane. However, mosquitoes reared from wild caught mosquito larvae or the progeny of wild-caught adult mosquitoes are a better representation of natural populations of anophelines in a given field site. Although there are indications for different oocysts densities in natural versus unnatural vectors [54], it is currently unclear if the choice of mosquitoes might influence conclusions on malaria transmission potential. Mosquito mortality prior to the dissection day was not reported for any of the studies we analyzed. If there is an association between gametocyte density and mosquito mortality, mosquito mortality may bias feeding results by artificially lowering the infectivity rate that is assessed in mosquitoes that survive until the day of dissection. Transmission reducing immune responses might reduce *Plasmodium*-induced mosquito mortality by reducing the number of ookinetes that penetrate the midgut epithelium [55], [56]; this phenomenon may be of particular relevance to future transmission blocking vaccines and transmission reducing interventions.

Lastly, all the data presented in this manuscript were based on the detection of oocysts on the mosquito midgut wall. This can be a very labour intensive endpoint. Development of high throughput molecular [57] or serological methods [58] to detect infected mosquitoes may replace the visual examination of midguts in transmission experiments. This would not only reduce the time needed to determine the results but may also allow for testing more mosquitoes per condition and improve statistical power. Investigation into these gaps in our understanding of parasite-vector-host interactions, factors intrinsic to each species individually, and relevance/importance of assay conduct methodology described above and summarized in Table 6, when combined with the recommendations for conduct of mosquito feeding experiments in Table 5, may allow us to more accurately predict field transmission using laboratory assays.

### Conclusions

Antimalarial drugs and vaccines that specifically target the transmission stages of malaria parasites are high on the priority list of the research agenda for malaria eradication [59], [60]. Initial screening may rely on *in vitro* or *ex vivo* systems where cultured parasites are used to test the gametocytocidal or transmission-reducing activities of drug and vaccine candidates [20], [61], [62], [63] but the evaluation of the most promising candidates will ultimately require assays that can quantify their impact on the infectiousness of individuals infected with naturally occurring parasite strains. In this report, we have provided a detailed overview of mosquito feeding assays in endemic regions. Based on the large amount of data we reviewed in this manuscript, we conclude that direct skin feeding assays are most sensitive to detect the human transmission potential to mosquitoes. Membrane feeding assays allow an approximation of this transmission potential; the association between both assays justifies the use of whole blood membrane feeding assays in settings where direct skin feeding is not possible and in studies where the gametocyte concentration of the mosquito blood meal or transmission reducing immune responses are of interest. Membrane feeding assays, in particular serum replacement assays, require optimization to maximize the potential of currently available tools.

## References

[1] Meis JF, Wismans PG, Jap PH, Lensen AH, Ponnudurai T (1992) A scanning electron microscopic study of the sporogonic development of Plasmodium falciparum in Anopheles stephensi. Acta Trop 50: 227–236. 1348599 10.1016/0001-706x(92)90079-d

[2] Boudin C, Olivier M, Molez JF, Chiron JP, Ambroise-Thomas P (1993) High human malarial infectivity to laboratory-bred Anopheles gambiae in a village in Burkina Faso. Am J Trop Med Hyg 48: 700–706. 8517489 10.4269/ajtmh.1993.48.700

[3] Schneider P, Bousema JT, Gouagna LC, Otieno S, van dV, et al (2007) Submicroscopic Plasmodium falciparum gametocyte densities frequently result in mosquito infection. Am J Trop Med Hyg 76: 470–474. 17360869

[4] Robert V, Awono-Ambene HP, Le Hesran JY, Trape JF (2000) Gametocytemia and infectivity to mosquitoes of patients with uncomplicated Plasmodium falciparum malaria attacks treated with chloroquine or sulfadoxine plus pyrimethamine. Am J Trop Med Hyg 62: 210–216. 10813475 10.4269/ajtmh.2000.62.210

[5] Graves PM (1980) Studies on the use of a membrane feeding technique for infecting Anopheles gambiae with Plasmodium falciparum. TransRSocTropMedHyg 74: 738–742. 10.1016/0035-9203(80)90189-3 7010696

[6] Ouedraogo AL, Bousema T, Schneider P, de Vlas SJ, Ilboudo-Sanogo E, et al (2009) Substantial contribution of submicroscopical Plasmodium falciparum gametocyte carriage to the infectious reservoir in an area of seasonal transmission. PLoS One 4: e8410. 20027314 10.1371/journal.pone.0008410 PMC2793432

[7] Mitri C, Thiery I, Bourgouin C, Paul RE (2009) Density-dependent impact of the human malaria parasite Plasmodium falciparum gametocyte sex ratio on mosquito infection rates. Proc Biol Sci 276: 3721–3726. 19656795 10.1098/rspb.2009.0962 PMC2817308

[8] Robert V, Read AF, Essong J, Tchuinkam T, Mulder B, et al (1996) Effect of gametocyte sex ratio on infectivity of Plasmodium falciparum to Anopheles gambiae. Trans R Soc Trop Med Hyg 90: 621–624. 9015496 10.1016/s0035-9203(96)90408-3

[9] Butcher GA (1997) Antimalarial drugs and the mosquito transmission of Plasmodium. Int J Parasitol 27: 975–987. 9363480 10.1016/s0020-7519(97)00079-9

[10] Kone A, van de Vegte-Bolmer M, Siebelink-Stoter R, van Gemert GJ, Dara A, et al (2010) Sulfadoxine-pyrimethamine impairs Plasmodium falciparum gametocyte infectivity and Anopheles mosquito survival. Int J Parasitol 40: 1221–1228. 20515695 10.1016/j.ijpara.2010.05.004 PMC3744324

[11] Hogh B, Gamage-Mendis A, Butcher GA, Thompson R, Begtrup K, et al (1998) The differing impact of chloroquine and pyrimethamine/sulfadoxine upon the infectivity of malaria species to the mosquito vector. Am J Trop Med Hyg 58: 176–182. 9502601 10.4269/ajtmh.1998.58.176

[12] Bousema T, Sutherland CJ, Churcher TS, Mulder B, Gouagna LC, et al (2011) Human immune responses that reduce the transmission of Plasmodium falciparum in African populations. Int J Parasitol 41: 293–300. 20974145 10.1016/j.ijpara.2010.09.008 PMC3052432

[13] Graves PM, Doubrovsky A, Sattabongkot J, Battistutta D (1992) Human antibody responses to epitopes on the Plasmodium falciparum gametocyte antigen PFS 48/45 and their relationship to infectivity of gametocyte carriers. Am J Trop Med Hyg 46: 711–719. 1377881 10.4269/ajtmh.1992.46.711

[14] Gamage-Mendis AC, Rajakaruna J, Carter R, Mendis KN (1992) Transmission blocking immunity to human Plasmodium vivax malaria in an endemic population in Kataragama, Sri Lanka. Parasite Immunol 14: 385–396. 1437231 10.1111/j.1365-3024.1992.tb00013.x

[15] Michel K, Kafatos FC (2005) Mosquito immunity against Plasmodium. Insect Biochem Mol Biol 35: 677–689. 15894185 10.1016/j.ibmb.2005.02.009

[16] Hallett RL, Dunyo S, Ord R, Jawara M, Pinder M, et al (2006) Chloroquine/sulphadoxine-pyrimethamine for gambian children with malaria: transmission to mosquitoes of multidrug-resistant Plasmodium falciparum. PLoS Clin Trials 1: e15. 16871318 10.1371/journal.pctr.0010015 PMC1513405

[17] Targett G, Drakeley C, Jawara M, von Seidlein L, Coleman R, et al (2001) Artesunate reduces but does not prevent posttreatment transmission of Plasmodium falciparum to Anopheles gambiae. J Infect Dis 183: 1254–1259. 11262208 10.1086/319689

[18] Mharakurwa S, Kumwenda T, Mkulama MA, Musapa M, Chishimba S, et al (2011) Malaria antifolate resistance with contrasting Plasmodium falciparum dihydrofolate reductase (DHFR) polymorphisms in humans and Anopheles mosquitoes. Proc Natl Acad Sci U S A 108: 18796–18801. 22065788 10.1073/pnas.1116162108 PMC3219121

[19] Lambrechts L, Halbert J, Durand P, Gouagna LC, Koella JC (2005) Host genotype by parasite genotype interactions underlying the resistance of anopheline mosquitoes to Plasmodium falciparum. Malar J 4: 3. 15644136 10.1186/1475-2875-4-3 PMC548507

[20] van der Kolk M, de Vlas S, Saul A, van de Vegte-Bolmer M, Eling WM, et al (2004) Evaluation of the standard membrane feeding assay (SMFA) for the determination of malaria transmission reducing activity using empirical data. Parasitology 130: 13–22. 10.1017/s0031182004006067 15700753

[21] van der Kolk M, de Vlas SJ, Sauerwein RW (2006) Reduction and enhancement of Plasmodium falciparum transmission by endemic human sera. Int J Parasitol 36: 1091–1095. 16790244 10.1016/j.ijpara.2006.05.004

[22] Bousema T, Drakeley C (2011) Epidemiology and infectivity of *Plasmodium falciparum* and *Plasmodium vivax* gametocytes in relation to malaria control and elimination. Clin Microbiol Rev 24: 377–410. 21482730 10.1128/CMR.00051-10 PMC3122489

[23] Bonnet S, Gouagna C, Safeukui I, Meunier JY, Boudin C (2000) Comparison of artificial membrane feeding with direct skin feeding to estimate infectiousness of Plasmodium falciparum gametocyte carriers to mosquitoes. Trans R Soc Trop Med Hyg 94: 103–106. 10748913 10.1016/s0035-9203(00)90456-5

[24] Boudin C, van der Kolk M, Tchuinkam T, Gouagna C, Bonnet S, et al (2004) Plasmodium falciparum Transmission Blocking Immunity under conditions of low and high endemicity in Cameroon. Parasite Immunol 26: 105–110. 15225297 10.1111/j.0141-9838.2004.00689.x

[25] Diallo M, Toure AM, Traore SF, Niare O, Kassambara L, et al (2008) Evaluation and optimization of membrane feeding compared to direct feeding as an assay for infectivity. Malar J 7: 248. 19055715 10.1186/1475-2875-7-248 PMC2640402

[26] Graves PM, Burkot TR, Carter R, Cattani JA, Lagog M, et al (1988) Measurement of malarial infectivity of human populations to mosquitoes in the Madang area, Papua New Guinea. Parasitology 96: 251–263. 3374964 10.1017/s003118200005825x

[27] Awono-Ambene HP, Diawara L, Robert V (2001) Comparison of direct and membrane feeding methods to infect Anopheles arabiensis with Plasmodium falciparum. Am J Trop Med Hyg 64: 32–34. 11425159 10.4269/ajtmh.2001.64.32

[28] Drakeley CJ, Eling W, Teelen K, Bousema JT, Sauerwein R, et al (2004) Parasite infectivity and immunity to Plasmodium falciparum gametocytes in Gambian children. Parasite Immunol 26: 159–165. 15367293 10.1111/j.0141-9838.2004.00696.x

[29] Mulder B, Lensen T, Tchuinkam T, Roeffen W, Verhave JP, et al (1999) Plasmodium falciparum: membrane feeding assays and competition ELISAs for the measurement of transmission reduction in sera from Cameroon. Exp Parasitol 92: 81–86. 10329369 10.1006/expr.1999.4398

[30] Tchuinkam T, Mulder B, Dechering K, Stoffels H, Verhave JP, et al (1993) Experimental infections of Anopheles gambiae with Plasmodium falciparum of naturally infected gametocyte carriers in Cameroon: factors influencing the infectivity to mosquitoes. Trop Med Parasitol 44: 271–276. 8134766

[31] Gouagna LC, Bonnet S, Gounoue R, Verhave JP, Eling W, et al (2004) Stage-specific effects of host plasma factors on the early sporogony of autologous Plasmodium falciparum isolates within Anopheles gambiae. Trop Med Int Health 9: 937–948. 15361106 10.1111/j.1365-3156.2004.01300.x

[32] Mulder B, Tchuinkam T, Dechering K, Verhave JP, Carnevale P, et al (1994) Malaria transmission-blocking activity in experimental infections of Anopheles gambiae from naturally infected Plasmodium falciparum gametocyte carriers. Trans R Soc Trop Med Hyg 88: 121–125. 8153987 10.1016/0035-9203(94)90534-7

[33] Bonnet S, Gouagna LC, Paul RE, Safeukui I, Meunier JY, et al (2003) Estimation of malaria transmission from humans to mosquitoes in two neighbouring villages in south Cameroon: evaluation and comparison of several indices. Trans R Soc Trop Med Hyg 97: 53–59. 12886806 10.1016/s0035-9203(03)90022-8

[34] Coulibaly MB (1999) Comparaison de deux méthodes d'infection expérimentale des moustiques comme moyens d'évaluation des vaccins de blocage de la transmission du paludisme à Bancoumana, Mali. PhD thesis University of Bamako, Mali.

[35] Gouagna LC (1999) Aspect biologique et immunologique du développement sporogonique de Plasmodium falciparum chez An. gambiae. PhD thesis Université de Yaoundé, Cameroon.

[36] Bland JM, Altman DG (1986) Statistical methods for assessing agreement between two methods of clinical measurement. Lancet 1: 307–310. 2868172

[37] DerSimonian R, Laird N (1986) Meta-analysis in clinical trials. Control Clin Trials 7: 177–188. 3802833 10.1016/0197-2456(86)90046-2

[38] Drakeley CJ, Jawara M, Targett GAT, Walraven G, Obisike U, et al (2004) Addition of artesunate to chloroquine for treatment of Plasmodium falciparum malaria in Gambian children causes a significant but short-lived reduction in infectiousness for mosquitoes. Trop Med Int Health 9: 53–61. 14728607 10.1046/j.1365-3156.2003.01169.x

[39] Sutherland CJ, Ord R, Dunyo S, Jawara M, Drakeley CJ, et al (2005) Reduction of Malaria Transmission to Anopheles Mosquitoes with a Six-Dose Regimen of Co-Artemether. PLoS Med 2: e92. 15839740 10.1371/journal.pmed.0020092 PMC1087200

[40] Chardome M, Janssen PJ (1952) Enquête sur l'incidence malarienne par la method dermique dans la region de Lubilash (Congo Belge). Ann Soc Belge Med Trop 32: 209–211. 12976890

[41] van den Berghe L, Chardome M, Peel E (1952) Supériorité des préparations de scarification du derme sur les préparations de sang périphérique pour le diagnostic de malaria. An Instit Med Trop 9: 553–562. 13058140

[42] Nacher M (2004) Does the shape of Plasmodium falciparum gametocytes have a function? Med Hypotheses 62: 618–619. 15050117 10.1016/j.mehy.2003.11.011

[43] Pichon G, Awono-Ambene HP, Robert V (2000) High heterogeneity in the number of Plasmodium falciparum gametocytes in the bloodmeal of mosquitoes fed on the same host. Parasitology 121: 115–120. 11085230 10.1017/s0031182099006277

[44] Sutherland CJ (2009) Surface antigens of Plasmodium falciparum gametocytes–a new class of transmission-blocking vaccine targets? Mol Biochem Parasitol 166: 93–98. 19450726 10.1016/j.molbiopara.2009.03.007

[45] Babiker HA, Schneider P, Reece SE (2008) Gametocytes: insights gained during a decade of molecular monitoring. Trends Parasitol 24: 525–530. 18801702 10.1016/j.pt.2008.08.001 PMC2764380

[46] Joice RC, Narasimhan V, Montgomery J, Seydel KB, Pierre-Louis W, et al.. (2011) Simultaneous Quantification of Asexual and Sexual Stages During Malaria Infection. Conference Abstract American Society of Tropical Medicine & Hygiene, Philadelphia.

[47] Seufi AM, Galal FH (2010) Role of Culex and Anopheles mosquito species as potential vectors of rift valley fever virus in Sudan outbreak, 2007. BMC Infect Dis 10: 65. 20222979 10.1186/1471-2334-10-65 PMC2841661

[48] World Health Organization (2010) MALVAC 2010 Scientific Forum: Measures of Efficacy of Anti-malarial Interventions Against Malaria Transmission, 15–16 November 2010. Geneva.

[49] Gouagna LC, Ferguson HM, Okech BA, Killeen GF, Kabiru EW, et al (2004) Plasmodium falciparum malaria disease manifestations in humans and transmission to Anopheles gambiae: a field study in Western Kenya. Parasitology 128: 235–243. 15074873 10.1017/s003118200300444x

[50] Drakeley CJ, Secka I, Correa S, Greenwood BM, Targett GA (1999) Host haematological factors influencing the transmission of Plasmodium falciparum gametocytes to Anopheles gambiae s.s. mosquitoes. Trop Med Int Health 4: 131–138. 10206267 10.1046/j.1365-3156.1999.00361.x

[51] Telang A, Frame L, Brown MR (2007) Larval feeding duration affects ecdysteroid levels and nutritional reserves regulating pupal commitment in the yellow fever mosquito Aedes aegypti (Diptera: Culicidae). J Exp Biol 210: 854–864. 17297145 10.1242/jeb.02715

[52] Mwangangi JM, Mbogo CM, Muturi EJ, Nzovua JG, Kabiru EW, et al (2007) Influence of biological and physicochemical characteristics of larval habitats on the body size of Anopheles gambiae mosquitoes (Diptera: Culicidae) along the Kenyan coast. J Vector Borne Dis 44: 122–127. 17722866 PMC2705333

[53] Gilles JR, Lees RS, Soliban SM, Benedict MQ (2011) Density-dependent effects in experimental larval populations of Anopheles arabiensis (Diptera: Culicidae) can be negative, neutral, or overcompensatory depending on density and diet levels. J Med Entomol 48: 296–304. 21485365 10.1603/me09209

[54] Harris C, Morlais I, Churcher TS, Awono-Ambene P, Gouagna LC, et al (2012) Plasmodium falciparum produce lower infection intensities in local versus foreign Anopheles gambiae populations. PLoS One 7: e30849. 22292059 10.1371/journal.pone.0030849 PMC3266902

[55] Ferguson HM, Read AF (2002) Why is the effect of malaria parasites on mosquito survival still unresolved? Trends Parasitol 18: 256–261. 12036738 10.1016/s1471-4922(02)02281-x

[56] Dawes EJ, Churcher TS, Zhuang S, Sinden RE, Basanez MG (2009) Anopheles mortality is both age- and Plasmodium-density dependent: implications for malaria transmission. Malar J 8: 228. 19822012 10.1186/1475-2875-8-228 PMC2770541

[57] Bell AS, Ranford-Cartwright LC (2004) A real-time PCR assay for quantifying Plasmodium falciparum infections in the mosquito vector. Int J Parasitol 34: 795–802. 15157762 10.1016/j.ijpara.2004.03.008

[58] Beier MS, Schwartz IK, Beier JC, Perkins PV, Onyango F, et al (1988) Identification of malaria species by ELISA in sporozoite and oocyst infected Anopheles from western Kenya. Am J Trop Med Hyg 39: 323–327. 3056055 10.4269/ajtmh.1988.39.323

[59] MalERA Consultative Group on Drugs (2011) A research agenda for malaria eradication: drugs. PLoS Med 8: e1000402. 21311580 10.1371/journal.pmed.1000402 PMC3026688

[60] MalERA Consultative Group on Vaccines (2011) A research agenda for malaria eradication: vaccines. PLoS Med 8: e1000398. 21311586 10.1371/journal.pmed.1000398 PMC3026701

[61] Tanaka TQ, Williamson KC (2011) A malaria gametocytocidal assay using oxidoreduction indicator, alamarBlue. Mol Biochem Parasitol 177: 160–163. 21316401 10.1016/j.molbiopara.2011.02.005 PMC3075389

[62] Buchholz K, Burke TA, Williamson KC, Wiegand RC, Wirth DF, et al (2011) A high-throughput screen targeting malaria transmission stages opens new avenues for drug development. J Infect Dis 203: 1445–1453. 21502082 10.1093/infdis/jir037 PMC3080890

[63] Peatey CL, Spicer TP, Hodder PS, Trenholme KR, Gardiner DL (2011) A high-throughput assay for the identification of drugs against late-stage Plasmodium falciparum gametocytes. Mol Biochem Parasitol 180: 127–131. 21939693 10.1016/j.molbiopara.2011.09.002

